# Plasma and Brain-Derived Extracellular Vesicle Biomarkers Following a Randomized Controlled Trial of Choline for Neurodevelopment in Fetal Alcohol Spectrum Disorder: A Pilot Analysis

**DOI:** 10.3390/nu18183022

**Published:** 2026-09-16

**Authors:** Phu V. Tran, Zia L. Maxim, Judith K. Eckerle, Steven H. Zeisel, Michael K. Georgieff, Jeffrey R. Wozniak

**Affiliations:** 1 Department of Pediatrics, University of Minnesota, Minneapolis, MN 55455, USA; matti252@umn.edu (Z.L.M.); ecke0056@umn.edu (J.K.E.); georg001@umn.edu (M.K.G.); 2 Masonic Institute for the Developing Brain, University of Minnesota, Minneapolis, MN 55455, USA; jwozniak@umn.edu; 3 Nutrition Research Institute, Gillings School of Global Public Health, University of North Carolina, Chapel Hill, NC 27599, USA; 4 Department of Psychiatry & Behavioral Sciences, University of Minnesota, Minneapolis, MN 55455, USA

**Keywords:** fetal alcohol spectrum disorders, choline, brain-derived extracellular vesicles, neuroinflammation, biomarkers, randomized controlled trial

## Abstract

Background: Postnatal choline supplementation improves memory, non-verbal IQ, executive function, and white-matter microstructure in children with fetal alcohol spectrum disorder (FASD) across randomized controlled trials; however, peripheral and brain biomarkers of these effects are uncharacterized. Methods: We measured eight immune and neurotrophic biomarkers (TNFα, transferrin receptor 1 [TfR1], BDNF, ferritin, MCP-1, RANTES, CRP, and Eotaxin) in plasma and brain-derived extracellular vesicles (BDEVs) from 24 children with FASD randomized to choline (*n* = 13; 7 male, 6 female) or placebo (*n* = 11; 7 male, 4 female), before and approximately nine months after treatment (23 with paired pre/post samples). Values were log_10_-transformed to correct for non-normal distribution. We modeled Group × Time effects with linear mixed models, adjusted for age, sex, and sample storage duration, cross-checked with cluster-robust ordinary least squares, and tested plasma-to-BDEV concordance for each analyte with linear regression adjusted for age and sex. Multiplicity was corrected by Benjamini–Hochberg FDR. Results: Plasma MCP-1 showed a nominally significant Group × Time interaction (*p* = 0.035, q = 0.28), rising over time in the placebo but not the choline group. Ferritin and CRP showed similar patterns that did not reach significance (interaction *p* = 0.099 and *p* = 0.118; q = 0.31 for both). RANTES and BDNF rose significantly in BDEVs over time, without evidence of a differential change by treatment group, and survived FDR correction (both q ≤ 0.02); ferritin also rose significantly (*p* = 0.021) but its Time effect did not survive FDR correction (q = 0.06). BDEV TfR1 was nominally lower in the choline group than placebo at both timepoints (*p* = 0.023; q = 0.13). Plasma levels were associated with BDEV levels for five markers (ferritin, TfR1, TNFα, Eotaxin, RANTES; all *p* < 0.05), independent of age, sex, time, and group; all five associations survived FDR correction (q ≤ 0.01). The plasma-BDEV association for MCP-1 became non-significant when adjusted for time and group (*p* = 0.134, q = 0.13). CRP and BDNF showed a between-person, but not within-person, plasma-BDEV association. Conclusions: In this small, exploratory sample of children with FASD, a condition characterized by brain iron deficiency and inflammation, choline was associated with an attenuated rise in pro-inflammatory plasma MCP-1 relative to placebo, generating a new hypothesized candidate biomarker for further study. Most BDEV markers changed over time (Pre-to-Post), not with treatment. Plasma and BDEV BDNF were not significantly associated overall, although a between-person association was present without a corresponding within-person signal, suggesting that inferring central nervous system function from plasma BDNF should proceed with caution. These hypothesis-generating findings support further, larger-scale investigation of BDEV content analysis as a candidate complement to this trial program’s cognitive and neuroimaging outcomes. Replication and expansion of the BDEV panel of analytes in a larger sample are needed. Trial registration: ClinicalTrials.Gov [NCT02735473]; registered 2 April 2016.

## 1. Background

Fetal alcohol spectrum disorder (FASD) is a common, under-recognized cause of lifelong neurodevelopmental impairment [1,2,3]. No biological treatment is approved for its core cognitive and behavioral deficits [4,5]. Choline is an essential nutrient with established roles in phospholipid synthesis, acetylcholine production, and one-carbon (methylation) metabolism [6,7]. In animal models, choline attenuates alcohol-related hippocampal cellular, molecular, and behavioral deficits [8,9,10] and, therefore, it has been investigated as a postnatal neurodevelopmental intervention in children with prenatal alcohol exposure [4,5,11,12].

This research program has run a series of randomized, double-blind, placebo-controlled trials in independent cohorts with very similar designs. Children received 9 months of daily choline or placebo, starting at ages 2.5–5.9 years. In the original efficacy trial, choline improved performance on a hippocampus-dependent elicited imitation (EI) memory task with the largest effect in younger children [5]. A pooled analysis of this trial and two later RCTs (*n* = 104) confirmed a trend-level choline advantage on EI sequential memory. This advantage was also age moderated. Neither analysis found an effect on IQ or executive function at 9 months [11]. At four-year follow-up, the choline group showed higher non-verbal IQ, better visual-spatial and non-verbal working memory scores, and fewer parent-reported ADHD symptoms than placebo [12]. At seven-year follow-up, the group differences had grown. They now included lower-order executive function (processing speed, color naming) and more coherent white matter microstructure (a lower orientation dispersion index in the splenium of the corpus callosum), seen only in the choline group [4]. A companion neurophysiological study found that event-related potential (ERP) markers of recognition memory (Nc amplitude, positive slow wave) correlated with EI memory performance at trial completion. ERP measures did not differ by treatment group, likely due to limited power in that subsample [13].

Together, these studies map the behavioral, cognitive, and structural neuroimaging correlates of postnatal choline supplementation in this population. However, no study to date has examined choline’s peripheral or central biological signatures, particularly those indexing the documented neuroinflammation and iron deficiency found in preclinical models [14,15].

Brain-derived extracellular vesicles (BDEVs) are small vesicles that cross the blood–brain barrier. Their cargo is thought to be enriched for proteins originating from neural and glial cells, though this has not been fully validated against direct, paired central nervous system sampling for the CNTN2-based enrichment method used here (see Materials and Methods) and in previous studies [16,17]. Measurement of analytes found in BDEVs may therefore offer a candidate, minimally invasive proxy for certain aspects of central nervous system biology and can be sampled from a simple phlebotomy procedure [16,17,18,19]. In this pilot analysis, we measured plasma and BDEV-normalized concentrations of eight markers: two iron-handling proteins (ferritin, transferrin receptor 1 [TfR1]), one neurotrophic factor (BDNF), four immune/inflammatory markers (TNFα, MCP-1, RANTES, CRP), and one chemokine (Eotaxin). We measured these before and 9 months after the choline/placebo supplementation. We had three aims: first, to test whether plasma or BDEV trajectories of these markers differ between choline and placebo groups; second, to test whether such differences persist after adjusting for age and sex, given that this developmental window includes rapid hippocampal and white matter maturation; and third, to test whether plasma and BDEV concentrations of the same analyte are related, a first step toward understanding what BDEV cargo adds beyond only plasma measurement of biomarkers.

## 2. Materials and Methods

### 2.1. Parent-Study Methods and Participants

This substudy drew on all children with FASD who took part in this research program’s randomized, double-blind, placebo-controlled choline trial (NCT02735473) [11]. Detailed methods for the parent trial (recruitment, Institute of Medicine diagnostic criteria, randomization, choline dosing and formulation, and blinding) were described previously in an earlier publication [11]. We summarized them only briefly here. Parents completed a comprehensive informed consent process. All procedures were approved by the University of Minnesota’s Institutional Review Board prior to study initiation. Choline was administered under the Federal Drug Administration Investigational New Drug (IND) application #107085. The trial was registered on ClinicalTrials.gov as NCT02735473 (2 April 2016) prior to enrollment of the first participant. The clinical trial protocol and statistical plan are available by request from the corresponding author. Children, aged 2.5 years to 5.9 years at the time of enrollment, were recruited from the University of Minnesota’s FASD diagnostic clinic as well as community FASD clinics and support groups for families raising children with FASD. Exclusion criteria were: other diagnosed neurodevelopmental disorders at the time of enrollment (e.g., Autism Spectrum Disorder, Down syndrome) except Attention-Deficit/Hyperactivity Disorder (ADHD), neurological conditions (e.g., epilepsy, traumatic brain injury), medical conditions impacting the brain, or known history of very low birthweight (less than 1500 g). ADHD was not exclusionary because it is very common in FASD [20]. Prenatal polysubstance exposure is also very common in FASD [21], and we had previously reported that treatment groups across each of the three RCTs did not differ on polysubstance exposure [11]. Therefore, we did not exclude participants for comorbid drug exposure. Briefly, participants were randomly assigned (1:1 allocation) to choline vs. placebo for 9 months; randomization tables were prepared ahead of the study by a blinded statistician. The Investigational Drug Services Pharmacy at the University of Minnesota supplied the study drug to participants in coded packets. Investigators and participants were blinded to the treatment group. The trial used a parent-administered supplement of choline or placebo given once daily over a 9-month span. The treatment duration was chosen to allow for sufficient developmental change that would be detectable by the outcome instruments. Participants received 500 mg daily of choline (1.25 g choline bitartrate) or a placebo. Parents combined the fruit-flavored drink mix containing choline or placebo with water and provided the drink to the child each day. Adherence was high (78% to 88% across studies) and has been described in a previous publication [11].

**Figure 1:**
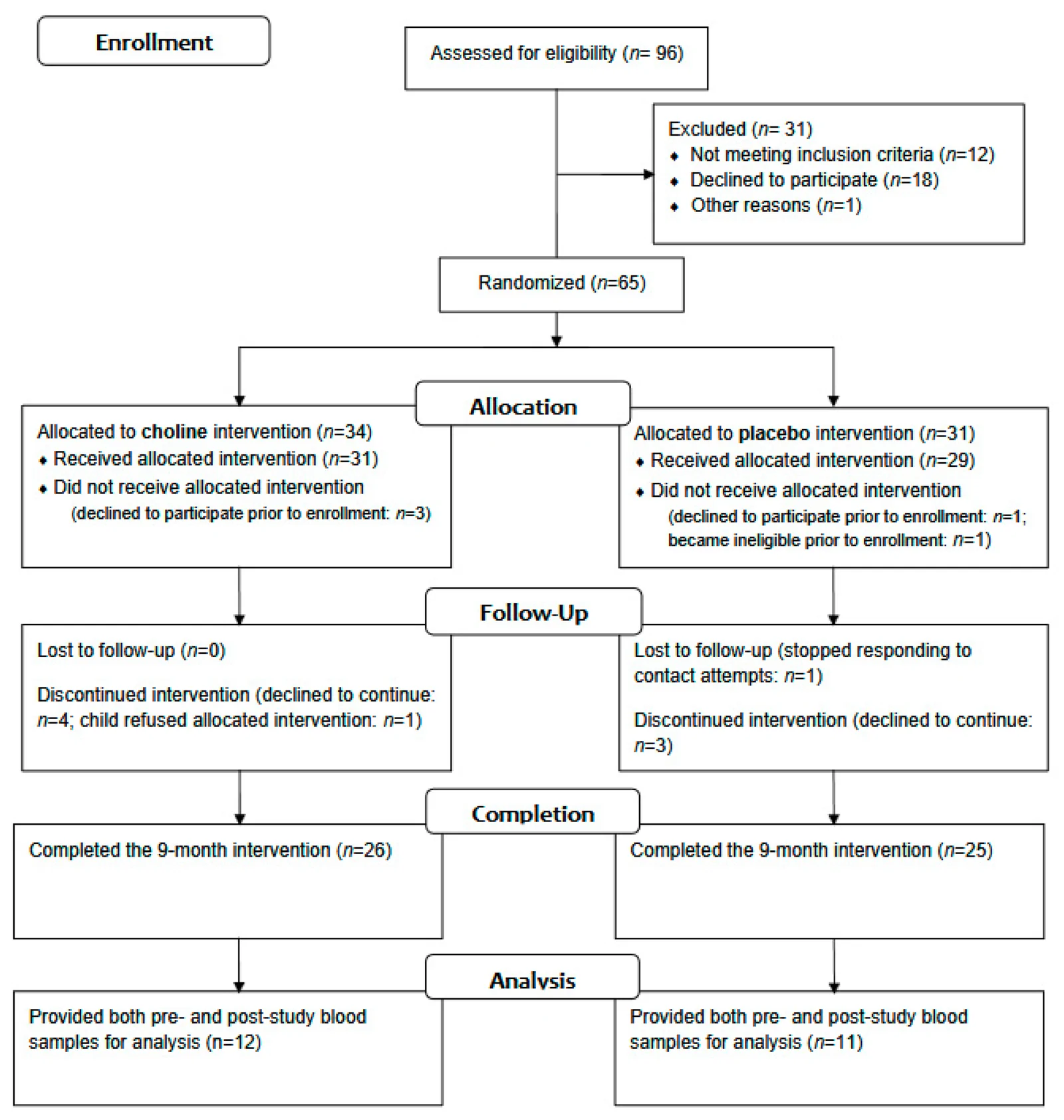
CONSORT flow diagram of participant enrollment, allocation, follow-up, and analysis. Flow of participants through this biomarker substudy, nested within the parent randomized, double-blind, placebo-controlled choline trial for FASD (NCT02735473). Numbers reflect the full parent-trial cohort through randomization and intervention; the Analysis boxes report the subset who provided both Pre- and Post-study blood samples for this substudy (*n* = 12 choline, *n* = 11 placebo); one additional choline group participant contributed a Pre-only sample and is included in the analysis sample described in Methods (total *n* = 13 choline, *n* = 11 placebo).

The analytic sample included the entire cohort, which consisted of 24 children ages 2.5 to 5.9 years at enrollment: 13 choline-treated (7 male, 6 female) and 11 placebo (7 male, 4 female). Blood was drawn at a pre-supplementation (“Pre”) visit. For 23 of the 24 children, blood was drawn again at a post-supplementation (“Post”) visit, nine months later. Table 1 summarizes sample characteristics; all 24 participants were drawn from this single parent trial (NCT02735473). Participant flow through enrollment, allocation, follow-up, and analysis is shown in the CONSORT diagram (Figure 1).

### 2.2. Biomarker Assessment

We measured TNFα, TfR1, BDNF, ferritin, MCP-1, RANTES, CRP, and Eotaxin at each visit in whole plasma (pg/mL per 10 µL) and in isolated BDEVs (pg per 10^8^ particles). We recorded BDEV particle concentration (particles/mL) and mean particle diameter (nm). This report followed the CNTN2-immunoprecipitation BDEV workflow used elsewhere in this research program (University of Minnesota, Masonic Institute for the Developing Brain) that had been validated in preclinical and clinical samples [22,23]. We used “BDEV” throughout as shorthand for CNTN2-immunoprecipitated vesicles from plasma. This enrichment strategy was designed to capture a CNTN2+, neural-enriched vesicle population, but it did not by itself establish that the captured particles are exclusively brain-derived. More precise terms such as CNTN2-enriched or CNTN2+ EVs, or putative BDEVs, would also be appropriate. We retained “BDEV” for brevity and for consistency with prior publications from this program that use the same convention [22,23]. In brief, the workflow had four steps. First, it isolated total small extracellular vesicles (sEVs) from plasma using a size-exclusion gravity-flow column (Exo-Spin 96, Cell Guidance Systems, St. Louis, MO, USA). Second, it enriched this sEV fraction for BDEVs by immunoprecipitation with an anti-human contactin-2 antibody (CNTN2, 1.5 µg/capture, PA5-101541, Invitrogen, Carlsbad, CA, USA). CNTN2 is a glycosyl-phosphatidyl-inositol-anchored neuronal adhesion protein with high central nervous system specificity [24,25,26,27,28], which makes it preferable to alternative capture targets such as L1CAM or NCAM, both of which show appreciable non-neural expression [29,30,31]. The workflow washed CNTN2-positive vesicles to remove unbound material, then eluted and neutralized them before −80 °C storage. Third, it quantified particle size and concentration in the BDEV-enriched preparation by nanoparticle tracking analysis (ZetaView MONO, Particle Metrix, Ammersee, Germany). As expected, this preparation showed smaller mean particle diameters than the total unenriched sEV fraction from the same sample (Supplemental Table S1). Fourth, it quantified protein cargo using magnetic bead-based multiplex immunoassays (Luminex platform, Discovery grade analytes, R&D Systems, Minneapolis, MN, USA). Each BDEV assay used a fixed particle input (1 × 10^8^ particles). Each corresponding whole-plasma assay used a fixed volume (10 µL), keeping the plasma and BDEV concentrations of a given marker on comparable, pre-specified inputs, rather than comparing across arbitrarily different sample amounts. Since BDEV analyte concentrations were normalized to a fixed particle input, a rise in a given BDEV analyte reflected higher cargo content per fixed particle input, not necessarily a rise in the total amount of that protein carried by circulating EVs. This distinction is relevant given that BDEV particle concentration itself changed over the study interval (see Section 3). Plasma was stored at −80 °C until use for BDEV isolation. BDEV isolates were also stored at −80 °C until assay. Paired Pre and Post samples from the same participant were run on the same assay plate/batch to minimize batch effects. Inter-assay coefficients of variation for the Luminex panel ranged from 0.54% to 3.16% with a grand average of 1.28% across all markers. No analyte value in this dataset fell below the assay’s lower limit of detection (LOD; values below the LOD would have been recorded as missing per protocol). Assay staff remained blinded to treatment-group assignment [22,23].

### 2.3. Statistical Analysis

Shapiro–Wilk testing showed non-normal distributions for 15 of the 16 plasma and BDEV analytes (compartments combined). Log_10_-transformation normalized 13 of these. Both raw and log_10_-scale results were reported.

Two complementary statistical approaches were used. First, linear mixed models (Group × Time, subject-level random intercept) tested for Group, Time, and Group × Time effects for the 16 analytes (both compartments) plus BDEV particle count (concentration) and size, adjusted for age, sex, and sample storage duration (Supplemental Table S2). Second, cluster-robust ordinary least squares (OLS; standard errors clustered by subject) cross-checked the mixed models and tested cross-compartment association (log_10_[BDEV] ~ log_10_[Plasma] + age + sex) for each analyte. Within-group (paired t-test/Wilcoxon) and between-group (Welch’s t-test/Mann–Whitney U) comparisons were run on log_10_ and raw scales.

Since 16 analytes were each tested across three model terms (Group, Time, Group × Time), Benjamini–Hochberg false-discovery-rate (FDR) correction was applied separately within each term and each compartment (six families of 8 tests; q-values were reported alongside nominal *p*-values). FDR correction was applied to these mixed models tests and separately to the cross-compartment regressions (two families of 8 tests: the initial pooled model and the Time/Group-adjusted refit). The within-/between-person decomposition of plasma-BDEV associations, and the BDEV particle concentration and size analyses were exploratory and were not corrected for multiplicity; results were reported as nominal *p*-values only.

All 16 mixed models used the full available data (47 observations, 24 subjects: 23 paired Pre/Post, 1 Pre-only [ID8283], no Post-visit blood draw). Residuals were normally distributed (Shapiro *p* > 0.10) for 14 of 16 models. Plasma MCP-1 (Shapiro *p* = 0.028) and plasma CRP (Shapiro *p* = 0.001) departed from normality, so nonparametric results were weighted heavily for these. For plasma MCP-1′s Group × Time interaction, a leave-one-subject-out sensitivity sweep kept *p*-values between 0.010 and 0.070 across all 24 iterations. Excluding the outlier noted in Supplemental Table S1 (ID7706) shifted the *p*-value from 0.030 to 0.034, indicating minimal impact. Models with a nominally significant or near-significant (*p* < 0.10) Time or interaction effect were refit using baseline (fixed) rather than visit-specific age; results were materially unchanged. With only 24 clusters, cluster-robust inference was interpreted cautiously and treated as secondary.

For each fixed-effect term in the mixed models (log_10_[analyte] ~ Group + Time + Group × Time + Age + Sex + storage duration, random intercept, REML; reference levels Placebo/Pre/Female), standardized effect size was computed as the coefficient divided by residual SD (Cohen’s d scale). Because each model included a Group × Time interaction term, the Time coefficient reflects the Pre-to-Post change in the placebo (reference) group, and the Group coefficient reflects the choline vs. placebo difference at the Pre (reference) visit. A non-significant Group × Time interaction does not establish that the longitudinal change was equivalent between groups. Group-specific Pre-to-Post estimates and 95% confidence intervals for each analyte were reported. For the cross-compartment regression, we reported R^2^ and Cohen’s f^2^ = R^2^/(1 − R^2^) along with a partial R^2^ and partial f^2^ isolating the plasma term’s incremental contribution ([R^2^ full − R^2^ without plasma]/[1 − R^2^ without plasma]). We also refitted each cross-compartment regression adding Time and Group, and separately decomposed plasma concentration into between-subject mean (each child’s average log_10_ plasma level across available visits) and a within-subject deviation to distinguish between-person from within-person plasma-BDEV associations. Full point estimates and 95% confidence intervals for all standardized effect sizes are provided in Supplemental Table S3. Analyses were performed in Python v3.11.15 (pandas 3.0.2, scipy 1.17.1, statsmodels 0.15.0, numpy 2.4.4).

## 3. Results

### 3.1. Age-, Sex-, and Storage-Duration-Adjusted Group × Time Effects

**Figure 2:**
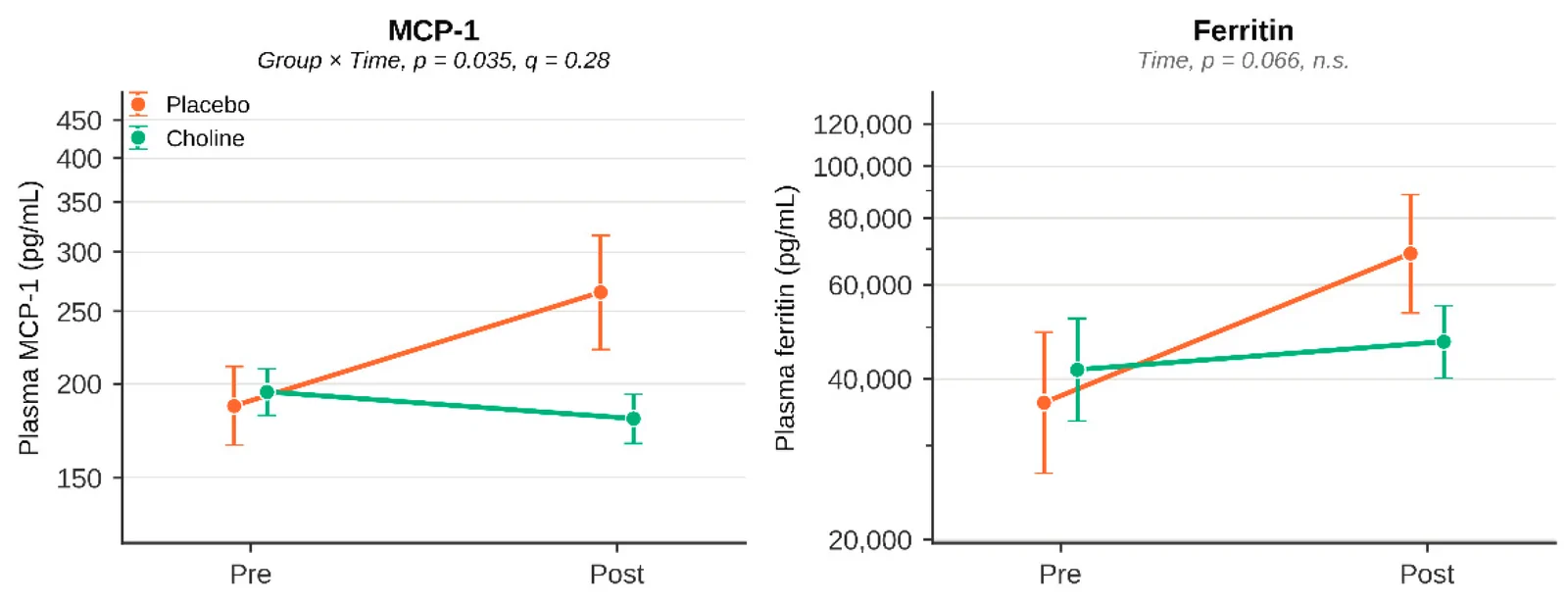
Pre-to-Post trajectories for plasma MCP-1 and ferritin, adjusted for age, sex, and storage duration. MCP-1 showed a nominally significant (*p* = 0.035) Group × Time interaction; ferritin’s Time effect did not reach nominal significance. Points and lines show the geometric mean (back-transformed from log_10_) at Pre and Post. Teal is the choline group; orange is placebo. Error bars are ±1 SE on the back-transformed log_10_ scale. Italicized text names the comparisons/interactions that reached *p* < 0.05.

In plasma, only MCP-1 showed a nominally significant Group × Time interaction (*p* = 0.035; q = 0.28), rising from Pre to Post more in the placebo group (median +18.6 pg/mL) than in the choline group (median +5.7 pg/mL) (Figure 2, left panel). Plasma ferritin showed a near nominal (non-significant) Time effect (*p* = 0.066, q = 0.37, Figure 2, right panel). Neither ferritin nor CRP showed evidence of a differential change by treatment group (Group × Time interaction *p* = 0.099 and *p* = 0.118, respectively). No other plasma analyte showed a Group, Time, or interaction effect. Storage duration was significantly and negatively associated with most plasma analyte levels (TNFα, TfR1, BDNF, MCP-1, RANTES, CRP; all q ≤ 0.024), except for ferritin and Eotaxin, consistent with degradation of some plasma proteins during long-term freezer storage.

**Figure 3:**
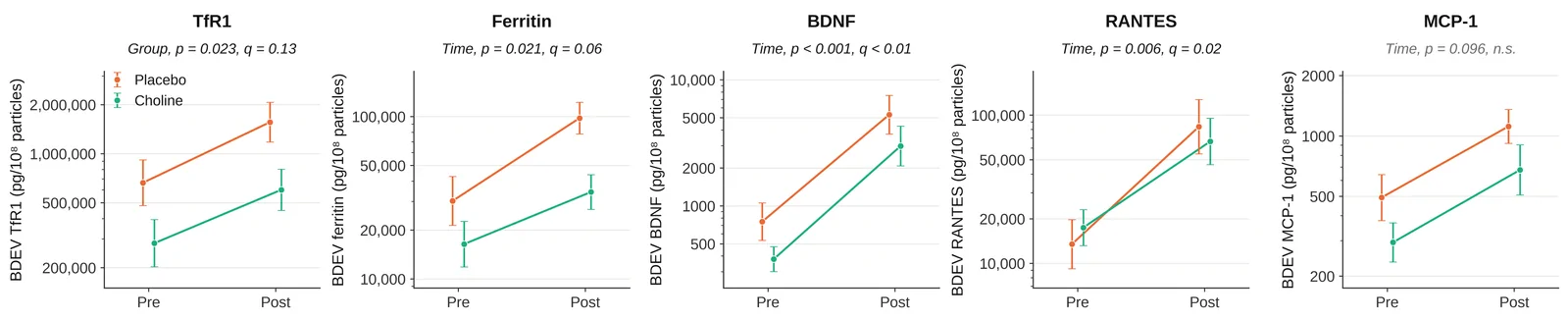
Pre-to-Post trajectories for BDEV analytes with a significant (*p* < 0.05) Group, Time, or Group × Time effect, adjusted for age, sex, and storage duration. MCP-1 is also shown for comparison; its Time effect did not reach nominal significance. *Y*-axis units are pg/10^8^ BDEV particles; axes are not shared across panels given the different concentration ranges among the analytes. Error bars are ±1 SE (log_10_ scale).

In BDEVs, the dominant pattern was a significant Time effect (Pre-to-Post increase in the placebo reference group) without evidence of a differential change by treatment group for any targeted BDEV analyte (Table 2). RANTES and BDNF rose significantly from Pre to Post and survived within-compartment FDR correction (RANTES *p* < 0.001, q < 0.01; BDNF *p* < 0.001, q < 0.01, Figure 3). Ferritin also rose significantly from Pre to Post (*p* = 0.021), but its Time effect did not survive FDR correction (q = 0.06). Eotaxin and TNFα showed similar, smaller rises that did not reach nominal significance (Eotaxin *p* = 0.072, q = 0.15; TNFα *p* = 0.139, q = 0.19). BDEV TfR1 showed a nominally significant Group effect (choline vs. placebo difference at the Pre reference visit; *p* = 0.023) that did not survive FDR correction (q = 0.13). Eotaxin and TNFα showed the same directional Group effect, also not significant after correction (*p* = 0.081, q = 0.13 and *p* = 0.071, q = 0.13, respectively). Follow-up cross-sectional comparisons showed that the BDEV TfR1 and TNFα group differences were present at both the Pre and Post visits, indicating a baseline between-group difference that persisted through the study rather than a treatment-induced change. No BDEV analyte showed a significant Group × Time interaction. Standardized Time-effect sizes for these BDEV markers were large (BDNF d = +1.81; RANTES d = +1.26; ferritin d = +1.10; MCP-1 d = +0.77), and the BDEV TfR1 Group effect was of similar magnitude (d = −0.94).

### 3.2. BDEV Particle Count and Size

The BDEV particle concentration and size analyses were exploratory and were not corrected for multiplicity. BDEV particle concentration showed a nominal Pre-to-Post decrease without evidence of a differential change by treatment group (age-, sex-, and storage duration-adjusted Time effect, *p* = 0.046). Mean particle diameter showed a non-significant Pre-to-Post increase over the same interval (*p* = 0.080). Neither measure showed a significant Group or Group × Time effect. Pre and Post values by treatment group are summarized in Supplemental Table S4. Storage duration was not a significant predictor of either measure (concentration *p* = 0.297; size *p* = 0.634).

### 3.3. Association Between Plasma and BDEV Levels of the Same Analyte

Table 3 shows the cross-compartment regression results. FDR correction was applied separately within the initial (unadjusted) and Time/Group-adjusted models (two families of 8 tests). In the initial age- and sex-adjusted model, which pooled Pre and Post observations, plasma concentration was associated with BDEV concentration of the same analyte for six markers: ferritin, TfR1, TNFα, Eotaxin, RANTES, and MCP-1 (all *p* < 0.05, all six survived FDR correction, q < 0.03; R^2^ range 0.15–0.36). Because this model could not separate within-child change from between-child differences, we refit each regression adding Time and Group. Ferritin, TfR1, TNFα, Eotaxin, and RANTES remained significant and survived FDR correction (all *p* < 0.01, q < 0.01). CRP became significant only in this Time/Group-adjusted model (*p* = 0.024, q = 0.03), despite not reaching nominal significance in the initial, unadjusted model. The plasma-BDEV association for MCP-1 became non-significant after adjustment for Time and Group (*p* = 0.134, q = 0.13). To separate within- from between-person coupling, plasma concentration of each analyte was further decomposed into each child’s between-subject mean and within-subject deviation from that mean. For ferritin, TfR1, TNFα, Eotaxin, and RANTES, both the within- and between-person plasma terms were independently associated with BDEV level (all *p* < 0.05, nominal). For CRP and BDNF, only the between-person term was nominally associated with BDEV level (CRP *p* = 0.002; BDNF *p* = 0.048), with no within-person signal (CRP *p* = 0.97; BDNF *p* = 0.72); the CRP association above therefore reflected between-person differences rather than within-person plasma-BDEV coupling. For MCP-1, neither component was significant (within *p* = 0.48; between *p* = 0.095). The f^2^ values above describe the full age/sex-adjusted model; Table 3 also reports a partial R^2^ and partial f^2^ isolating the plasma term’s incremental contribution.

## 4. Discussion

This pilot analysis examined plasma and BDEV immune and neurotrophic biomarkers in children from a postnatal choline supplementation trial. Plasma MCP-1 was the only marker with a different Pre-to-Post trajectory by treatment group; after adjustment for age, sex, and storage duration, the choline group showed a smaller rise over time than the placebo group. This finding was nominal (*p* = 0.035) and did not survive BH-FDR correction for multiplicity within the plasma panel (q = 0.28), and therefore should be read as one hypothesis-generating signal among many comparisons in this exploratory pilot, not as confirmatory evidence of a treatment effect. MCP-1 (CCL2) is a chemokine involved in monocyte recruitment and neuroinflammatory signaling [32]. An attenuated Pre-to-Post rise in MCP-1 under choline would fit two independent lines of evidence for a choline-MCP-1 link. First, in a mouse model, dietary choline deficiency raised colonic MCP-1 after infectious challenge. It also raised Eotaxin and RANTES, both part of our panel. Choline-sufficient and choline-excess diets did not raise these markers [33]. Second, choline is the metabolic precursor of acetylcholine. Acetylcholine is the efferent signal of the cholinergic anti-inflammatory pathway through which vagal/nicotinic-receptor signaling suppresses macrophage cytokine release [34]. This single-marker association in a small exploratory human cohort is best viewed as hypothesis-generating and not evidence that either mechanism operated here. Additional confirmatory studies are needed.

While MCP-1 is a pro-inflammatory marker, human and animal studies have tied it to memory and cognitive performance. Except for a single childhood study, most of this evidence comes from aging and neurodegenerative research. In a longitudinal study of 79 preterm infants who received blood transfusion during hospital stay, of whom 26 completed cognitive evaluation at 12 months of age, higher plasma MCP-1 was inversely associated with neurocognitive score, particularly in female infants [35]. In a longitudinal study of 399 older adults, rising plasma MCP-1 predicted declining verbal memory over roughly two years. The association was specific to memory; it did not extend to other cognitive domains tested [36]. In Alzheimer’s disease and mild cognitive impairment, higher plasma MCP-1 tracked with greater baseline severity and faster two-year cognitive decline [37]. In a preclinical study, injecting CCL2 (MCP-1) directly into the rat hippocampus impaired spatial memory and object-recognition performance, accompanied by altered inflammatory, glutamate-metabolism, and apoptosis-related gene expression in the same brain region [38]. Collectively, this literature suggests MCP-1 as a plausible mechanistic bridge between peripheral inflammation and hippocampal memory function, beyond a marker of general inflammation. Considered alongside the MCP-1 literature described above, this research program’s previously reported cognitive measures (i.e., Elicited Imitation memory, IQ, and working memory [5,11,12]) suggest that the present finding raises a testable hypothesis. Plasma MCP-1 trajectories may be a peripheral correlate of this cohort’s memory outcomes. Further confirmation is needed in a future analysis of an independent and sufficiently powered cohort that links biomarker and cognitive data from the same visits.

The more consistent finding across the panel was a significant Time effect in the BDEV compartment (Pre-to-Post increase in the placebo reference group), without evidence of a differential change by treatment group for any BDEV analyte. Adjusted for age, sex, and storage duration, RANTES and BDNF rose significantly and survived FDR correction. Ferritin also rose significantly but its Time effect narrowly missed the FDR threshold (q = 0.06), requiring a more cautious interpretation than the RANTES and BDNF findings. BDEV particle concentration fell over the same interval, without a significant change in mean particle size (both exploratory analyses, uncorrected for multiplicity). This developmental window, ages 4 to 5 years in this cohort, overlaps with a period of rapid hippocampal and white-matter maturation. This trial program’s findings have emphasized that period throughout, including the corpus callosum microstructural changes reported at long-term follow-up [4]. These BDEV trajectories reflect a longitudinal (Pre-to-Post) change without evidence of a differential change by treatment group, rather than indicating a treatment-specific effect. This design cannot distinguish age/developmental maturation from other time-related processes, including pre-analytical factors such as storage duration.

BDEV TfR1 showed a Group effect, reflecting the choline vs. placebo difference at the Pre reference visit (nominal *p* = 0.023, q = 0.13), and BDEV TNFα and Eotaxin showed the same pattern without reaching nominal significance (*p* = 0.071 and *p* = 0.081). However, this difference was already present at the Pre visit. Despite randomization, this trial program has noted baseline group imbalances before. For example, unexpected racial-composition differences between arms emerged at long-term follow-up [4]. The present finding is best described as a baseline characteristic of this small sample, not a consequence of treatment.

Ferritin and TfR1 were the two most consistently altered analytes in this panel. Both are central to cellular iron handling. Ferritin is the primary intracellular iron-storage protein [39,40]. TfR1 is the receptor that mediates cellular iron uptake and typically indexes iron need [41]. Iron is required for myelination, monoamine synthesis, and hippocampal energy metabolism during this same early-childhood window [42]. Early iron deficiency has well-documented, sometimes persistent, effects on infant neurodevelopment [43]. Measuring these two analytes also continues prior work from this research program. BDEV cargo, including BDNF along with the CNTN2 capture target used to enrich for BDEVs, has previously been proposed as a candidate marker of neonatal iron-deficiency risk in cord blood [44]. Given that brain iron deficiency can be a contributing factor for FASD neurodevelopmental deficits [45,46], analysis of both peripheral and central iron status may offer potential mechanistic insights. However, we cannot tell, from this dataset alone, whether the ferritin and TfR1 trajectories reflect iron status specifically, more general BDEV cargo turnover, or both. Classical peripheral iron indices, such as hemoglobin, serum iron, and transferrin saturation, were not available for comparison.

A statistically significant plasma-BDEV association was not detected for BDNF overall; in the within/between-person decomposition, only the between-person plasma term was nominally associated with BDEV BDNF (*p* = 0.048), with no within-person signal (*p* = 0.72). However, BDEV BDNF showed the strongest Time effect of any marker in the panel. Choline’s proposed neurodevelopmental mechanisms include hippocampal effects on synaptogenesis and cholinergic signaling [5,12]. BDNF has also been proposed to play a role in this cohort’s previously reported memory and executive-function outcomes [5,12,13]. Prior work has proposed that BDEV-packaged BDNF could reflect local (neuronal/glial) regulation, distinct from circulating plasma BDNF [22,47,48]; plasma BDNF has sometimes been used as a proxy for brain BDNF under the assumption that it reflects central levels [49,50,51,52]. Neither CSF nor brain tissue was sampled in this cohort, so that assumption remains untested. A non-significant overall plasma-BDEV association for BDNF in this small sample (*n* = 24) does not establish independent or compartment-specific CNS regulation. A future analysis could relate BDEV and plasma BDNF directly to this cohort’s published EI memory, IQ, and white matter outcomes.

This pattern is worth comparing to a separate BDEV study in untreated first-episode psychosis. BDNF and CRP are the two analytes measured in both cohorts [22]. In that study, BDNF, CRP, and S100B showed no significant serum-to-BDEV correlations, in either diagnostic group. BDEV levels of BDNF and CRP were significantly lower in psychosis, despite unchanged serum levels of the same proteins. We interpreted this as evidence that BDEV cargo indexes CNS-proximal, actively regulated vesicular pathways, not passive uptake or leakage of peripheral protein into circulating vesicles [22]. Applied here, this offers one plausible explanation. Six of eight analytes showed plasma-BDEV associations, but not for BDNF or CRP specifically. Circulating CRP is produced almost exclusively by hepatocytes, as part of a systemic acute-phase response shaped by metabolic and environmental exposures [53,54,55]. It does not readily cross the blood–brain barrier outside of substantial inflammatory states [22]. Thus, CRP found within BDEVs may reflect locally regulated cargo sorting, not transfer of peripheral CRP into circulating vesicles. BDNF is similarly central to synaptic plasticity and hippocampal signaling. Prior studies reported positive cerebrospinal fluid and plasma BDNF correlations under certain conditions [51,52]. Still, a regulated, CNS-proximal secretory pathway, one that governs which BDNF reaches BDEV cargo, distinct from whatever sets bulk plasma BDNF concentration, would fit the pattern seen in both this cohort and the psychosis cohort. It should be noted that this is, at most, converging circumstantial evidence. It came from two small, non-overlapping studies in very different populations: early-childhood choline supplementation versus adolescent/young-adult first-episode psychosis. Thus, this was not a formal replication, but could be viewed as independent confirmation of our work and other studies [22,47]. Testing the underlying mechanism directly would need dedicated, paired plasma-BDEV-CSF sampling, which can be modeled in non-human primates.

## 5. Limitations

This analysis has several important limitations. The sample is small, consisting of 13 on choline, 11 on placebo. Age and Time are partially confounded by design. Children were necessarily older at the Post visit than at the Pre visit, in both arms. Adjusting for age helps but cannot fully separate developmental maturation from supplementation duration. Sample collection spanned rolling enrollment from July 2016 to February 2020, and within-subject Pre samples were necessarily collected before Post samples (median inter-visit gap 279 days; range 179–357). Pre samples plausibly carried more freezer time by the time each subject’s paired samples were assayed together; thus, residual confounding between age, time, and storage duration cannot be excluded. Storage duration was independently associated with lower levels of multiple plasma analytes (TNFα, TfR1, BDNF, MCP-1, RANTES, CRP; all q < 0.024) and, more selectively, lower BDEV CRP (q = 0.02) and, nominally, lower BDEV RANTES (q = 0.09), consistent with some degree of analyte degradation over long-term storage. While CNTN2 is enriched in the central nervous system, its expression is also found in the cardiac Purkinje fiber network [56,57]. Peripheral release of CNTN2+ EVs is possible but likely a minor contributor relative to CNS release; such release would dilute rather than fabricate brain-derived signals. Multiplicity correction further constrains the significant findings summarized above. After FDR correction, applied separately within each compartment, only two BDEV Time effects (BDNF and RANTES) survived correction; the plasma MCP-1 Group × Time interaction and the BDEV MCP-1 and TfR1 findings did not, and should be treated as nominal, hypothesis-generating signals rather than confirmed effects. Plasma MCP-1 and plasma CRP departed from normality on residual diagnostics, so their mixed-model results were cross-checked against nonparametric paired/independent-sample tests; agreement between the two approaches was reassuring, but neither fully substitutes for a larger, confirmatory sample. Cluster-robust standard errors, used as a secondary check on the mixed models, should be interpreted cautiously with only 24 clusters. With at most two time points per participant, the within/between-person decomposition of plasma-BDEV associations could not fully separate developmental change from measurement or batch effects, even though it distinguished within- from between-child sources of covariation. The CNTN2-based BDEV enrichment method had not been validated against direct, paired central nervous system tissue or cerebrospinal fluid sampling in this cohort, which was not ethically feasible in this pediatric RCT; the extent to which CNTN2-captured vesicles were exclusively brain-derived therefore remains an assumption rather than a demonstrated fact. Finally, all participants were drawn from a single parent trial, which limits the generalizability of these findings to the broader choline-FASD literature.

## 6. Conclusions

This was a small, exploratory biomarker analysis nested within a postnatal choline supplementation trial. Choline was associated with an attenuated rise in plasma MCP-1, relative to placebo, generating a new hypothesized biomarker for further confirmatory study. MCP-1 has an established link to memory function in human and animal populations, which makes this finding a promising candidate for direct testing against this cohort’s own cognitive outcomes. No BDEV analyte showed a significant Group × Time interaction; the BDEV compartment instead showed significant longitudinal (Pre-to-Post) change without evidence of a differential change by treatment group, most robustly for RANTES and BDNF after FDR correction. BDEV ferritin also rose significantly, but its Time effect narrowly missed the FDR threshold and should be read with more caution. Plasma and BDEV levels of the same analyte remained associated for most markers studied after accounting for time and treatment group, with the exception of MCP-1 and BDNF. Although these findings are preliminary, they suggest that peripheral and BDEV biomarkers are candidate biomarkers for future studies of choline-related neurodevelopmental outcomes in children with FASD. Taken together, these hypothesis-generating findings point to the exploratory MCP-1 signal and longitudinal changes in CNTN2-enriched EV cargo as candidates for further study, to be tested in larger, adequately powered cohorts, including associations with cognitive and neuroimaging outcomes.

## Figures and Tables

**Table 1:** Sample characteristics by treatment group.

Characteristic	Choline (*n* = 13)	Placebo (*n* = 11)	Total (N = 24)
Sex, male/female	7/6	7/4	14/10
Age at Pre-draw, y, mean (SD)	4.50 (0.90)	4.11 (1.07)	4.33 (0.99)
Age at Post-draw, y, mean (SD)	5.25 (0.85) ^a^	4.90 (1.08) ^a^	5.08 (0.98) ^a^
Subjects with paired Pre/Post samples, *n*	12	11	23

^a^ Age at Post is reported only for the 23 participants with a paired Post visit due to a missing Post-visit sample.

**Table 2:** Age-, sex-, and storage duration-adjusted linear mixed-model results (log_10_ scale) for plasma and BDEV analytes, with Benjamini–Hochberg FDR correction applied separately within each compartment and model-based Pre-to-Post changes (with 95% CI) for each treatment group.

Compartment	Analyte	Placebo Pre→Post Δ (95% CI)	Choline Pre→Post Δ (95% CI)	Group *p*	Group q	Group d	Time *p*	Time q	Time d	Group × Time *p*	Group × Time q	Group × Time d
**Plasma**	BDNF	1.06 [0.42, 2.63]	0.59 [0.25, 1.40]	0.143	0.57	+0.70	0.905	0.90	+0.06	0.312	0.62	−0.59
	CRP	1.10 [0.66, 1.82]	0.67 [0.41, 1.09]	0.100	0.57	+0.89	0.725	0.89	+0.17	0.118	0.31	−0.92
	Eotaxin	1.18 [0.77, 1.82]	1.21 [0.80, 1.82]	0.700	0.96	−0.18	0.450	0.89	+0.35	0.940	0.99	+0.04
	Ferritin	1.66 [0.97, 2.86]	1.00 [0.60, 1.68]	0.836	0.96	+0.14	0.066	0.37	+0.98	0.099	0.31	−0.97
	MCP-1	1.31 [0.96, 1.78]	0.85 [0.63, 1.15]	0.815	0.96	+0.10	0.094	0.37	+0.77	0.035 *	0.28	−1.23
	RANTES	0.78 [0.27, 2.27]	0.77 [0.28, 2.14]	0.217	0.58	+0.57	0.647	0.89	−0.21	0.993	0.99	−0.01
	TfR1	0.97 [0.90, 1.04]	0.99 [0.92, 1.06]	0.991	0.99	+0.01	0.440	0.89	−0.41	0.731	0.99	+0.20
	TNFα	1.07 [0.67, 1.70]	1.09 [0.70, 1.69]	0.709	0.96	−0.19	0.781	0.89	+0.13	0.949	0.99	+0.04
**BDEVs**	BDNF	6.78 [2.62, 17.53]	7.52 [3.06, 18.49]	0.123	0.14	−0.66	<0.001 *	<0.01	+1.81	0.867	0.99	+0.10
	CRP	1.10 [0.23, 5.32]	1.05 [0.24, 4.65]	0.081	0.13	−0.73	0.902	0.90	+0.06	0.962	0.99	−0.03
	Eotaxin	1.87 [0.94, 3.73]	1.63 [0.85, 3.13]	0.081	0.13	−0.89	0.073	0.15	+0.86	0.742	0.99	−0.19
	Ferritin	2.52 [1.15, 5.53]	1.57 [0.74, 3.32]	0.085	0.13	−0.86	0.021 *	0.06	+1.10	0.338	0.99	−0.56
	MCP-1	1.83 [0.90, 3.71]	1.83 [0.93, 3.59]	0.100	0.13	−0.74	0.096	0.15	+0.77	0.995	0.99	+0.00
	RANTES	4.24 [1.52, 11.77]	2.63 [1.00, 6.92]	0.723	0.72	+0.15	0.006 *	0.02	+1.26	0.477	0.99	−0.42
	TfR1	1.67 [0.67, 4.18]	1.50 [0.63, 3.59]	0.023 *	0.13	−0.94	0.274	0.31	+0.50	0.864	0.99	−0.10
	TNFα	1.89 [0.81, 4.38]	1.68 [0.75, 3.73]	0.071	0.13	−0.90	0.139	0.19	+0.70	0.823	0.99	−0.13

* *p* < 0.05. Since each model includes a Group × Time interaction term, Group *p* is the choline-vs-placebo difference at the Pre (reference) visit and Time *p* is the Pre-to-Post change in the Placebo (reference) group. Group × Time *p* is whether the Pre-to-Post change differed by treatment group. q is the Benjamini–Hochberg FDR-corrected *p*-value, computed separately within each compartment (Plasma, BDEVs) for each term (Group, Time, Group × Time). d is the standardized effect size for that term (fixed-effect coefficient ÷ model residual SD), on the same scale as Cohen’s d; positive values indicate higher levels in the choline group (Group), at the Post visit (Time), or a larger Post-Pre rise in the choline vs. placebo group (Group × Time). Placebo/Choline Pre→Post Δ (95% CI) is the model-based geometric-mean change from Pre to Post for each treatment group, back-transformed from the log_10_-scale Time (Placebo) or Time + Group × Time (Choline) coefficients; values above 1 indicate a rise from Pre to Post, values below 1 a fall. All 16 compartment-specific models adjust for age, sex, and storage duration covariate with a subject-level random intercept; *n* = 47 observations from 24 subjects (23 paired, 1 Pre-only).

**Table 3:** Cross-compartment regression: BDEV concentration as a function of plasma concentration, adjusted for age and sex.

Analyte	Plasma Slope (log_10_–log_10_)	*p* (Unadjusted)	q (Unadjusted)	*p* (Time/Group- Adjusted)	q (Time/Group- Adjusted)	R^2^ (f^2^)	Partial R^2^ (Partial f^2^)
**Ferritin**	0.78	<0.001 *	<0.01	<0.001 *	<0.01	0.36 (f^2^ = 0.55)	0.31 (f^2^ = 0.44)
**TNFα**	0.93	<0.001 *	<0.01	<0.001 *	<0.01	0.33 (f^2^ = 0.49)	0.27 (f^2^ = 0.37)
**Eotaxin**	0.75	0.004 *	0.01	0.006 *	0.01	0.25 (f^2^ = 0.33)	0.17 (f^2^ = 0.21)
**TfR**	3.02	0.005 *	0.01	0.005 *	0.01	0.15 (f^2^ = 0.18)	0.10 (f^2^ = 0.11)
**RANTES**	0.39	0.019 *	0.03	0.005 *	0.01	0.24 (f^2^ = 0.32)	0.16 (f^2^ = 0.18)
**MCP-1**	0.89	0.021 *	0.03	0.134	0.13	0.19 (f^2^ = 0.23)	0.14 (f^2^ = 0.17)
**CRP**	0.62	0.109	0.12	0.024 *	0.03	0.17 (f^2^ = 0.21)	0.06 (f^2^ = 0.06)
**BDNF**	0.20	0.287	0.29	0.068	0.08	0.07 (f^2^ = 0.08)	0.03 (f^2^ = 0.03)

* *p* < 0.05. Slope is the log_10_(BDEV)-on-log_10_(plasma) regression coefficient from an age- and sex-adjusted linear model, with standard errors clustered by subject; R^2^ (f^2^) reflects this model. Because this analysis pools repeated Pre and Post observations, each model was refit, adding Time and Group as covariates; the Time/Group-adjusted *p* and partial R^2^ (partial f^2^) columns reflect the plasma predictor in that refit model. f^2^ = R^2^/(1 − R^2^), Cohen’s effect-size index for the plasma predictor (≥0.02 small, ≥0.15 medium, ≥0.35 large, by convention). Benjamini–Hochberg FDR correction was applied separately within the unadjusted and Time/Group-adjusted models (two families of 8 tests); q is the FDR-corrected *p*-value for each.

## Data Availability

The dataset analyzed in the current study is available from the corresponding author on reasonable request.

## Supplementary Materials

The following supporting information can be downloaded at: https://www.mdpi.com/article/10.3390/nu18183022/s1, Supplemental Table S1: Total EV and BDEV Concentration and Size, Pre vs. Post Collection. Supplemental Table S2. Effect of a sample storage-duration covariate on BDEV particle concentration/size and on the eight BDEV and eight plasma analyte mixed models. Supplemental Table S3: Standardized effect sizes (Cohen’s d-equivalent) with 95% confidence intervals, from age-, sex-, and storage duration-adjusted mixed models. Supplemental Table S4: BDEV particle concentration and size, Pre and Post, by treatment group.

## Abbreviations

BDEV, brain-derived extracellular vesicle; BDNF, brain-derived neurotrophic factor; CRP, *C*-reactive protein; EI, elicited imitation; FASD, fetal alcohol spectrum disorder; MCP-1, monocyte chemoattractant protein-1; PAE, prenatal alcohol exposure; RANTES, regulated upon activation, normal T cell expressed and secreted (CCL5); sEV, small extracellular vesicle; TfR1, transferrin receptor 1; TNFα, tumor necrosis factor alpha.
