# Supplementary material for: Plasma and Brain-Derived Extracellular Vesicle Biomarkers Following a Randomized Controlled Trial of Choline for Neurodevelopment in Fetal Alcohol Spectrum Disorder: A Pilot Analysis
Source: Nutrients. 2026 Sep 16;18(18):3022. doi: 10.3390/nu18183022 (PMC13610065; doi:10.3390/nu18183022)
Supplement: Supplementary file 1 [file nutrients-18-03022-s001.zip › nutrients-4524840-supplementary.pdf]

Supplemental Table S1: Total EV and BDEV Concentration and Size, Pre vs. Post Collection.

Pre-collection (n = 24)

Sample ID	Total EV concentration (particles/mL)	Total EV diameter (nm)	BDEV concentration (particles/mL)	BDEV diameter (nm)
7596	100.0 x 10^9	91.8	11.0 x 10^9	96.2
7674	240.0 x 10^9	95.2	28.0 x 10^9	96.2
7698	82.0 x 10^9	105.0	15.0 x 10^9	95.4
7702	150.0 x 10^9	127.2	25.0 x 10^9	111.9
7706	170.0 x 10^9	122.5	23.0 x 10^9	89.3
7756	71.0 x 10^9	114.0	9.3 x 10^9	92.8
7839	130.0 x 10^9	143.8	9.4 x 10^9	52.9
7840	170.0 x 10^9	143.6	25.0 x 10^9	82.4
7841	140.0 x 10^9	118.1	26.0 x 10^9	94.4
8048	160.0 x 10^9	99.2	21.0 x 10^9	74.9
8228	87.0 x 10^9	131.6	30.0 x 10^9	86.2
8283	110.0 x 10^9	148.4	87.0 x 10^9	82.8
8383	70.0 x 10^9	93.2	23.0 x 10^9	63.2
8472	110.0 x 10^9	136.8	26.0 x 10^9	90.3
8598	120.0 x 10^9	135.1	25.0 x 10^9	99.0
8648	120.0 x 10^9	159.0	24.0 x 10^9	77.8
8650	120.0 x 10^9	158.9	12.0 x 10^9	94.3
8673	110.0 x 10^9	91.1	13.0 x 10^9	101.4
9024	140.0 x 10^9	129.0	9.0 x 10^9	95.9
9092	110.0 x 10^9	137.3	24.0 x 10^9	91.5
9214	130.0 x 10^9	166.4	27.0 x 10^9	108.7
9316	140.0 x 10^9	120.6	29.0 x 10^9	85.8
9420	110.0 x 10^9	153.1	3.7 x 10^9	88.9
9506	110.0 x 10^9	125.2	21.0 x 10^9	74.7
**Mean ± SD (n=24)**	**125.0 ± 36.8 x 10^9**	**126.9 ± 22.7**	**22.8 ± 15.6 x 10^9**	**88.6 ± 13.2**

Post-collection (n = 23)

Sample ID	Total EV concentration (particles/mL)	Total EV diameter (nm)	BDEV concentration (particles/mL)	BDEV diameter (nm)
7596	140.0 x 10^9	159.3	6.1 x 10^9	103.1
7674	200.0 x 10^9	127.2	8.3 x 10^9	76.5
7698	110.0 x 10^9	139.4	9.3 x 10^9	91.9
7702	120.0 x 10^9	143.8	18.0 x 10^9	117.3
7706	91.0 x 10^9	139.8	79.0 x 10^9	112.6

Sample ID	Total EV concentration (particles/mL)	Total EV diameter (nm)	BDEV concentration (particles/mL)	BDEV diameter (nm)
7756	120.0 x 10^9	159.1	12.0 x 10^9	103.8
7839	120.0 x 10^9	125.6	8.5 x 10^9	99.7
7840	110.0 x 10^9	180.8	7.2 x 10^9	91.2
7841	160.0 x 10^9	86.0	25.0 x 10^9	103.8
8048	72.0 x 10^9	150.6	7.5 x 10^9	88.1
8228	190.0 x 10^9	125.4	12.0 x 10^9	115.6
8383	80.0 x 10^9	146.2	12.0 x 10^9	89.0
8472	110.0 x 10^9	158.1	7.4 x 10^9	98.0
8598	84.0 x 10^9	169.4	10.0 x 10^9	71.9
8648	87.0 x 10^9	177.9	8.2 x 10^9	105.2
8650	91.0 x 10^9	200.9	12.0 x 10^9	97.8
8673	58.0 x 10^9	123.9	6.5 x 10^9	99.9
9024	160.0 x 10^9	137.2	3.7 x 10^9	100.5
9092	140.0 x 10^9	161.4	6.5 x 10^9	119.7
9214	120.0 x 10^9	139.1	7.8 x 10^9	110.1
9316	77.0 x 10^9	154.6	13.0 x 10^9	123.4
9420	72.0 x 10^9	151.1	6.8 x 10^9	114.1
9506	67.0 x 10^9	147.9	6.5 x 10^9	91.3
**Mean ± SD (n=23)**	**112.1 ± 38.7 x 10^9**	**148.0 ± 23.4**	**12.8 ± 15.1 x 10^9**	**101.1 ± 13.2**

Note: Sample 8283 has no matched Post sample. Sample 7706 shows an outlying BDEV concentration in both collections.

Supplemental Table S2. Effect of a sample storage-duration covariate on BDEV particle concentration/size and on the eight BDEV and eight plasma analyte mixed models.

Supplemental Table S2a. BDEV particle concentration and size, with and without a storage-duration covariate.

Outcome	Time effect, no storage covariate (coef, p)	Time effect, storage-adjusted (coef, p)	Storage-duration effect (coef, p)	R^2, no storage → with storage	Conclusion
BDEV particle concentration (log_{10} particles/mL)	−0.239, p=0.007	−0.206, p=0.028	+0.004/mo, p=0.256	0.35 → 0.37	Time effect attenuated but remains nominally significant; storage duration itself not a significant predictor.
BDEV particle size (nm)	+10.82, p=0.079	+10.06, p=0.099	−0.10/mo, p=0.660	0.21 → 0.21	Time effect was already borderline (p=0.079) and remains so; storage duration not a significant predictor.

Cluster-robust (subject-clustered) OLS models of the form outcome ~ Group + Time + Group×Time + Age + Sex, refit with an added Storage covariate. Coefficients are for the Time term (log_{10} particles/mL per level, or nm) and for the added Storage term. Neither outcome's storage-duration coefficient reached nominal significance (both p>0.25); both Time effects persisted qualitatively after adjustment.

Supplemental Table S2b. Plasma analyte mixed models with and without a storage-duration covariate.

Plasma Analyte	Time p (original)	Time q (original)	Time p (storage-adjusted)	Time q (storage-adjusted)	Storage-duration effect (coef/mo, p)	Storage q
BDNF	0.345	0.459	0.905	0.905	−0.021*, p=0.005*	0.013*
CRP	0.120	0.321	0.725	0.892	−0.017*, p=<0.001*	0.004*
Eotaxin	0.227	0.438	0.450	0.892	−0.005, p=0.150	0.171
Ferritin	0.035*	0.139	0.066	0.375	−0.001, p=0.843	0.843
MCP-1	0.019*	0.139	0.094	0.375	−0.005*, p=0.018*	0.024*
RANTES	0.776	0.776	0.647	0.892	−0.023*, p=0.008*	0.016*
TfR1	0.618	0.706	0.440	0.892	−0.003*, p=0.002*	0.008*
TNFα	0.274	0.438	0.781	0.892	−0.010*, p=0.016*	0.024*

*Linear mixed models of the form analyte ~ Group + Time + Group×Time + Age + Sex, random intercept per subject (REML), refit with an added Storage covariate. p and q are for the Time term; each FDR family (original Time, storage-adjusted Time, Storage effect) is computed by Benjamini-Hochberg correction across all 8 plasma analytes, separately. No plasma Time effect was FDR-significant either before or after adjustment (all q≥0.14); ferritin and MCP-1 are the two analytes whose Time effect was nominally significant (*p<0.05) before adjustment and no longer reached even nominal significance*

afterward. The plasma MCP-1 Group × Time interaction (p=0.030, q=0.24) was essentially unchanged after storage adjustment (p=0.036, q=0.28).

Supplemental Table S2c. BDEV analyte mixed models (Table 2 methodology), with and without a storage-duration covariate.

BDEV Analyte	Time p (original)	Time q (original)	Time p (storage-adjusted)	Time q (storage-adjusted)	Storage-duration effect (coef/mo, p)	Storage q
BDNF	<0.001	<0.001	<0.001	<0.001	−0.006, p=0.396	0.633
CRP	0.393	0.393	0.902	0.902	−0.033, p=0.002*	0.017*
Eotaxin	0.052	0.083	0.073	0.147	−0.001, p=0.869	0.869
**Ferritin**	0.010	0.026	0.021	**0.056**	−0.003, p=0.619	0.825
MCP-1	0.035	0.071	0.096	0.154	−0.008, p=0.163	0.325
RANTES	<0.001	0.003	0.006	0.023	−0.016*, p=0.022*	0.086
TfR1	0.141	0.161	0.274	0.313	−0.010, p=0.119	0.317
TNFα	0.098	0.130	0.139	0.186	−0.002, p=0.749	0.856

*Linear mixed models of the form analyte ~ Group + Time + Group×Time + Age + Sex, random intercept per subject (REML), refit with an added Storage covariate. p and q are for the Time term; the two Time-effect FDR families (original, storage-adjusted) and the Storage-effect FDR family are each computed as Benjamini-Hochberg correction across all 8 analytes, separately. Group and Group×Time terms were not materially affected by storage adjustment for any analyte. *p<0.05. Ferritin (shaded) is the one analyte whose Time effect crosses the FDR threshold (q=0.03 → q=0.06) after storage adjustment; RANTES and BDNF, the other two originally FDR-significant analytes, remain significant after adjustment. CRP shows a nominally significant association with storage duration itself (p=0.002, q=0.02 across the Storage-effect family) but its Time effect is unaffected because it was never significant for Time.*

***Note:** Six of the eight plasma analytes (TNFα, TfR1, BDNF, MCP-1, RANTES, CRP) showed a significant negative association with estimated storage duration after FDR correction (all q≤0.024), consistent with degradation of some plasma proteins over long-term freezer storage, in contrast to the general stability of the BDEV-encapsulated analytes. This asymmetry is itself a notable, hypothesis-generating observation.*

Supplemental Table S3: Standardized effect sizes (Cohen's d-equivalent) with 95% confidence intervals, from age-, sex-, and storage duration-adjusted mixed models

Effect sizes correspond to the p-values reported in Table 2 and are derived from the same models (fixed-effect coefficient ÷ model residual SD, $\log_{10}$ scale). Positive values indicate higher levels in the choline group (Group term), at the Post visit (Time term), or a larger Post–Pre rise in the choline vs. placebo group (Group × Time term).

Compartment	Analyte	Group d [95% CI]	Time d [95% CI]	Group × Time d [95% CI]
Plasma	MCP-1	+0.10 [-0.76, +0.96]	+0.77 [-0.13, +1.67]	-1.23 [-2.38, -0.08]
Plasma	Ferritin	+0.14 [-1.18, +1.45]	+0.98 [-0.06, +2.02]	-0.97 [-2.12, +0.18]
Plasma	CRP	+0.89 [-0.17, +1.95]	+0.17 [-0.79, +1.13]	-0.92 [-2.07, +0.23]
Plasma	BDNF	+0.70 [-0.23, +1.63]	+0.06 [-0.86, +0.98]	-0.59 [-1.74, +0.56]
Plasma	TfR	+0.01 [-1.32, +1.34]	-0.41 [-1.46, +0.63]	+0.20 [-0.95, +1.36]
Plasma	RANTES	+0.57 [-0.34, +1.48]	-0.21 [-1.13, +0.70]	-0.01 [-1.15, +1.14]
Plasma	TNFα	-0.19 [-1.21, +0.82]	+0.13 [-0.81, +1.08]	+0.04 [-1.11, +1.19]
Plasma	Eotaxin	-0.18 [-1.10, +0.74]	+0.35 [-0.56, +1.27]	+0.04 [-1.11, +1.19]
BDEVs	Ferritin	-0.86 [-1.84, +0.12]	+1.10 [+0.16, +2.03]	-0.56 [-1.71, +0.59]
BDEVs	RANTES	+0.15 [-0.68, +0.98]	+1.26 [+0.37, +2.16]	-0.42 [-1.56, +0.73]
BDEVs	Eotaxin	-0.89 [-1.89, +0.11]	+0.86 [-0.08, +1.80]	-0.19 [-1.35, +0.96]
BDEVs	TNFα	-0.90 [-1.87, +0.08]	+0.70 [-0.23, +1.63]	-0.13 [-1.28, +1.02]
BDEVs	TfR	-0.94 [-1.76, -0.13]	+0.50 [-0.39, +1.39]	-0.10 [-1.25, +1.05]
BDEVs	BDNF	-0.66 [-1.51, +0.18]	+1.81 [+0.91, +2.70]	+0.10 [-1.05, +1.25]
BDEVs	CRP	-0.73 [-1.55, +0.09]	+0.06 [-0.83, +0.95]	-0.03 [-1.17, +1.12]
BDEVs	MCP-1	-0.74 [-1.61, +0.14]	+0.77 [-0.14, +1.67]	+0.00 [-1.15, +1.15]

CI, confidence interval. Confidence intervals are wide given the small, unequal group sizes (choline n=13, placebo n=11) typical of this pilot analysis and should be interpreted as imprecise, hypothesis-generating estimates rather than precise population effect sizes.

Supplemental Table S4: BDEV particle concentration and size, Pre and Post, by treatment group.

Group	Visit	n	Concentration (×10⁹ particles/mL) Mean ± SD	Concentration Median [IQR]	Diameter (nm) Mean ± SD	Diameter Median [IQR]
All	Pre	24	22.8 ± 15.6	23.5 [12.8–26.0]	88.6 ± 13.2	90.9 [82.7–96.0]
All	Post	23	12.8 ± 15.1	8.3 [7.0–12.0]	101.1 ± 13.2	100.5 [91.6–111.4]
Choline	Pre	13	25.9 ± 20.1	24.0 [15.0–28.0]	88.3 ± 9.9	89.3 [85.8–95.4]
Choline	Post	12	15.9 ± 20.6	8.8 [7.3–12.3]	102.5 ± 13.0	102.2 [96.5–113.0]
Placebo	Pre	11	19.1 ± 7.1	21.0 [11.5–25.0]	89.0 ± 16.9	92.8 [78.7–97.6]
Placebo	Post	11	9.3 ± 3.6	7.8 [6.9–11.0]	99.5 ± 13.8	99.7 [91.3–107.0]

n = number of subjects with a valid measurement at that visit (Pre n=24, Post n=23; one subject [ID 8283] has a Pre-only sample, no Post visit). IQR, interquartile range (25th–75th percentile). Values are drawn from the same BDEV particle concentration and diameter measurements listed by subject in Supplemental Table 1. Subject ID 7706 is a concentration and diameter outlier (Post concentration 79.0 $\times 10^9$ particles/mL, more than 3-fold the next-highest Post value); excluding this subject changes the mean Pre-to-Post concentration change from −7.2 ± 15.7 to −10.1 ± 7.7 $\times 10^9$ particles/mL (n=22) and the mean diameter change from +12.2 ± 17.2 to +11.7 ± 17.4 nm (n=22), consistent with the sensitivity analyses reported in Results.
